# Association of vitamin B1/B6/B12 supplementation with sphingosine-1-phosphate signaling and its receptors in multiple sclerosis patients: relevance to LISPR1 and APOA1-AS

**DOI:** 10.1042/BSR20260065

**Published:** 2026-05-21

**Authors:** Noha A. Mehana, Heba R. Ghaiad, Mohammed M. Nooh, Mai A. Amer, Lobna Talaat El-Ghoneimy, Maheera H. Safwat

**Affiliations:** 1 Biochemistry Department, Faculty of Pharmacy, Cairo University, Cairo, Egypt; 2 Microbiology and Immunology Department, Faculty of Pharmacy, October University for Modern Sciences and Arts (MSA), Giza, Egypt; 3 Neurology Department, Faculty of Medicine, Cairo University, Cairo, Egypt

**Keywords:** lncRNA APOA1-AS, lncRNA LISPR1, multiple sclerosis, S1P, S1PR1, sphingosine kinases

## Abstract

MS is a lifelong autoimmune disorder striking the central nervous system (CNS). Despite the currently used disease-modifying therapies, patients are exposed to persistent neuropathy, pinpointing the need for supportive therapy. Neurotropic vitamins B1, B6, and B12 have been used to offer relief from immunological and neurological MS manifestations. The present study aimed to provide some mechanistic insights into the relationship of B1/B6/B12 vitamin supplementation with the development of MS regarding lipid metabolism and epigenetics. In this cross-sectional observational study, blood samples were obtained from 53 MS patients, including 25 patients, who had received daily vitamin B1/B6/B12 supplementation for over six months and 28 patients without supplementation. Plasma sphingosine-1-phosphate (S1P) and S1P receptor-1 (S1PR1) levels, lipid profile, and gene expression of ApoA1, sphingosine kinases 1&2 (SPHK1&2), S1PR1, as well as the lncRNAs APOA1-AS and LISPR1 were evaluated. Gene ontology and KEGG pathway enrichment analyses were conducted. Vitamin B1/B6/B12 supplementation was associated with a more favorable lipid profile. Supplemented patients also exhibited higher ApoA1 and lower APOA1-AS expressions compared with non-supplemented patients. Additionally, vitamin B1/B6/B12 supplementation was associated with lower expression levels of SPHK1, SPHK2, LISPR1, and S1PR1 and reduced circulating S1P concentrations. These findings imply significant associations between long-term vitamin B1/B6/B12 supplementation and alterations in lipid-related markers and sphingosine-associated signaling in MS patients. However, the observational design, selection bias, and small sample size limit causal inference and may not fully capture the heterogeneity of MS population. Besides, supplement adherence was self-reported and not objectively verified, and circulating vitamin levels were not measured.

## Background

Multiple sclerosis (MS) is a prevailing autoimmune disorder impacting the brain and spinal cord, distinguished by inflammatory alterations, disturbance of the blood-brain barrier (BBB), demyelination, microglial activation, and neuronal deterioration [1,2]. The etiology of the disease, although still inexplicable, is reputed to be a blend of genetic predisposition and a multitude of environmental elements [3]. MS is considered a major cause of disability among adults, with around three times higher incidence in women than men [4]. Individuals are usually affected after the age of thirty, initially by a relapsing-remitting phase, which commonly transforms gradually into the secondary-progressive type marked by deterioration of neurological function [5,6].

The pathology of the disease is triggered by autoreactive CD4 T lymphocytes that penetrate the BBB into the central nervous system (CNS), where they initiate an immune reaction specifically against myelin, which is further exacerbated by macrophages and glial cells, thus creating focal lesions designated by demyelination, inflammation, and neuronal degeneration [6].

Many disease-modifying therapies (DMTs) are currently used for management of MS; however, most MS patients still suffer from recurrent episodes, persistent painful symptoms, and medication side effects. Accordingly, the application of supportive/adjunct therapy might be beneficial in enhancing drug efficiency, alleviating patients’ neuropathy, and enhancing their quality of life in the presence of such a debilitating disease [7,8].

It has been well established that B vitamins, especially B1/B6/B12, are essential for the development and function of CNS [9] and play a major role in myelin restoration and preservation, axonal protection, and attenuation of neuroinflammation [10,11]. Especially, vitamins B6 and B12 have proven to affect MS incidence, pathogenesis, and progression and are commonly used to maintain immunological and neurological homeostasis in MS patients [12]. Thus, our study is directed to provide insights into the underlying mechanisms for the possible protective role of vitamin B1/B6/B12 combination against MS pathogenesis.

In that regard, two main axes of MS pathogenesis are investigated: the first is ApoA1/APOA1-AS and the second is S1PR1/S1P/LISPR1, in addition to the related sphingosine kinases SPHK1 and SPHK2. Apoprotein A1 (ApoA1) is the most abundant part of high-density lipoprotein (HDL) [13]. It is a constitutive anti-inflammatory molecule mainly through immune cell regulation [14]. ApoA1 can regulate immune cells via inhibition of immune cells generation, such as monocytes, macrophages, dendritic cells, neutrophils, and T lymphocytes [15]; decreasing the expression of related factors of immune cells such as vascular cell adhesion molecule-1 (VCAM-1), monocyte chemotactic protein 1 (MCP-1), and macrophage inflammatory protein 1 (MIP-1) [16]; inhibiting the synergistic effect of immune cells interactions, such as macrophage interactions with T-cells [17]; and altering the expression of ApoA1 in immune cells, including macrophages and monocytes, thus affecting their functions [18,19].

Recently, advances in immunometabolism show that ApoA1 can also regulate metabolic pathways within immune cells, especially lipid metabolism, mitochondrial activity, and cholesterol efflux, modulating inflammatory signaling and immune cell activation [14]. Through these immunological and metabolic regulatory mechanisms, ApoA1 could counteract the inflammatory and autoimmune processes associated with MS [20]. Additionally, reduced ApoA1 levels have been correlated with MS progression and disease severity [21–23].

ApoA1 levels are further modulated by the endogenously expressed long noncoding natural antisense transcript, lncRNA apolipoprotein A-1 antisense RNA (APOA1-AS), which has been shown to function as a negative transcriptional regulator of ApoA1, acting both *in vitro* and *in vivo* [24]. Recent advances in lncRNA biology have shown that lncRNAs are key epigenetic factors that regulate gene expression through different mechanisms, including chromatin remodeling, transcriptional interference, RNA–protein interactions, and modulation of metabolic and inflammatory pathways [25]. Dysregulation of lncRNA expression is associated with immune cell activation, lipid metabolism, and neuroinflammation involved in MS progression [26]. Therefore, targeting lncRNA-mediated regulatory networks, including APOA1-AS, to enhance ApoA1 expression may represent a promising therapeutic strategy for promoting neuronal protection and improving disease outcomes in MS.

Sphingosine-1-phosphate (S1P) is a critical lipid messenger of utmost importance in MS pathogenesis. S1P is mainly produced through ceramides hydrolysis inside lysosomes followed by phosphorylation by SPHK1 or SPHK2 [27]. S1P acts through different subtypes of G-protein-coupled receptors [28,29]. By binding to sphingosine-1-phosphate receptor-1 (S1PR1) residing on CCR7+ naïve, central memory B, and T cell lymphocytes, S1P modulates the migration of these cells from peripheral lymphoid organs, thus boosting inflammatory immune cell trafficking into the CNS [30,31]. Therefore, S1P receptor modulation constitutes a useful therapeutic target in MS to maintain CNS protection, reduce astrogliosis, and promote remyelination [32]. Several S1P receptor modulators, including fingolimod, siponimod, ozanimod, and ponesimod, are approved for MS treatment by inducing S1PR1 antagonism, thus preventing lymphocyte egress from lymph nodes [33]. Furthermore, S1P-targeted therapies have been reported to exert direct neuroprotective actions within CNS, such as astrogliosis reduction, regulation of microglial activation, and induction of remyelination [34].

Long intergenic noncoding RNA antisense to sphingosine-1-phosphate receptor-1 (lncRNA LISPR1) has been reported to regulate S1PR1 expression in endothelial cells, hence impacting S1P signaling. LISPR1 expression was found to be implicated in disease states in humans such as chronic obstructive pulmonary disease (COPD) [35]. According to our information, no study has yet observed the role of lncRNA LISPR1—via its effect on S1PR1 signaling—in MS pathogenesis.

On that account, the purpose of this research was to scrutinize the impact of long-term B1/B6/B12 vitamin complex supplementation on lipid metabolism and the two main axes implicated in MS pathogenesis: ApoA1/ APOA1-AS axis and S1PR1/ S1P /LISPR1 axis, in addition to the related SPHK1 and SPHK2.

## Study population

### Sample size calculation

Initially, G*Power software version 3.1.9.7 was applied to estimate sample size before the start of the study (*a priori*). We employed the two-tailed t test; a large effect size = 0.8, two independent groups, type I error α = 0.05, and type II error β = 0.2. Based on these assumptions, a minimum sample size of 26 per group yielded an actual power of 0.8074866 (approximately 80.75%).

### Multiple sclerosis patients

The present study comprised 53 MS patients, who visited Kasr Al-Ainy Multiple Sclerosis Research Unit (KAMSU), Cairo University Hospitals, Egypt. The medical history of all the patients was recorded, and they went through the conventional MS checkup, including physical and neurological examination, baseline brain and spinal cord magnetic resonance imaging (MRI), and standard laboratory tests to preclude any other possible diagnoses.

All patients participating in the study were selected based on a confirmed clinical diagnosis in accordance with the clinical and laboratory diagnostic criteria endorsed by the 2017 revision of the McDonald criteria [36]. Patients were classified depending on the disease course into the 4 clinical subtypes according to the classification of Lublin et al. [37]. Reported comorbidities were limited to non-metabolic conditions not expected to significantly influence lipid metabolism. Patients were excluded from the study for the presence of any of the following criteria: pregnancy, current or recent inflammatory or infectious diseases, familial hypercholesterolemia, antihyperlipidemic drugs, or steroid consumption within one month prior to enrollment.

In addition, patients receiving sphingosine-1-phosphate receptor modulators (e.g., fingolimod) were excluded due to their direct effects on S1P signaling pathways, which could interfere with the interpretation of the studied molecular markers. However, patients receiving other DMTs were not excluded or stratified during recruitment and thus represent a heterogeneous treatment population.

To study the effect of vitamin B supplementation on various biochemical parameters, MS patients recruited for the present study were divided into 2 groups. The first is vitamin B supplement group (*n* = 25), where MS patients were prescribed by their attending physician to receive an oral daily dose of 40 mg of benfotiamine, which is a synthetic, fat-soluble, S-acyl derivative of thiamine or vitamin B1, in addition to 60 mg of pyridoxine or vitamin B6 and 250 μg of cyanocobalamin or vitamin B12 (Eva group limited, Egypt).

The dose of vitamin B supplementation was prescribed by the attending physician according to the standard clinical dosing regimen typically used in such cases, with the aim of alleviating the patient’s presenting symptoms. MS patients with vitamin B supplementation have been receiving vitamin B1/B6/B12 supplements for at least a 3-month duration. The second group is “no supplement” MS group (*n* = 28), where MS patients were not prescribed by their attending physician to receive any vitamin B supplements. Adherence to vitamin supplementation was assessed based on patient self-report during clinical follow-up and was not objectively verified.

## Methods

### Sample collection

During their clinical visit, patients provided peripheral whole blood samples, which were collected into Vacuette collection tubes (Greiner Bio-One, Frickenhausen, Germany) containing EDTA. Centrifugation of the samples was carried out at 3000 × ***g*** for 15 min followed by separation of the buffy coats, which were instantaneously utilized for extraction of total RNA. The separated plasma was fractionated into aliquots and kept at −20°C.

#### Biochemical analysis

ApoA1 plasma levels were assayed by sandwich ELISA (NOVA, Beijing, China) and stated as nanograms per milliliter as per the manufacturers’ instructions. Total triglyceride and total cholesterol levels were determined by enzymatic spectrophotometric methods (Spectrum, Cairo, Egypt), and HDL cholesterol by precipitant method (Spectrum, Cairo, Egypt). Lastly, Friedewald’s formula was employed for assay of low-density lipoprotein (LDL) cholesterol concentrations [38].

Plasma S1P and S1PR1 levels were determined by ELISA kits obtained from MyBioSource (CA, U.S.A.) and expressed as nanograms per milliliter as per the manufacturers’ instructions.

#### Reverse transcription-quantitative polymerase chain reaction (RT-qPCR)

The expression levels of long non-coding RNAs; APOA1-AS and LISPR1, as well as the expression levels of SPHK1, SPHK2, and S1PR1 genes, were quantified via RT-qPCR.

##### Total RNA isolation

The total RNA content was isolated from the buffy coats using TRIzol Plus RNA Purification Kit (Invitrogen Life Technologies) as previously outlined. In brief, a mixture of 250 μl buffy coat with 750 μl TRIzol reagent was incubated for 10 min to allow total separation of nucleoproteins, followed by addition of 200 μl of chloroform (Sigma–Aldrich) and vortexing for 30 s [39]. The mixture was then left to stand for 5 min and centrifuged for 15 min at 12000 × ***g*** at 4°C for separation of aqueous and organic phases.

The aqueous phase was cautiously recovered and mixed with an equal volume of 70% ethanol (Sigma–Aldrich). Purification of the collected RNA was carried out by PureLink RNA Mini kit (Invitrogen Life Technologies), followed by washing one time with 700 µl Wash Buffer I and twice with 500 µl Wash Buffer II. The RNA was recovered in 30 μl of RNase-free water. The RNA concentration and quality were estimated via Q5000 UV-Vis Spectrophotometer (Quawell).

##### Reverse transcription and q-PCR

For reverse transcription, Quantitect Reverse Transcription kit (Qiagen) was used on the same day of RNA recovery. StepOne Real-Time PCR System (Thermo Fisher Scientific) and Maxima SYBR Green/ROX qPCR Master Mix (Thermo Fisher Scientific) were utilized for evaluation of gene expression. Glyceraldehyde-3-phosphate dehydrogenase (GAPDH) was utilized as the housekeeping reference gene. The oligonucleotides used for amplification were pre-designed using NCBI Primer-Blast and confirmed by *in silico* PCR tool of UCSC genome browser, and finally custom-made by Invitrogen and are listed in Supplementary Table S1.

In the thermal cycler, the following protocol was applied: initial enzyme activation for 10 min at 95°C followed by 45 cycles of denaturation for 15 s at 95°C, annealing for 30 s at 60°C, and extension for 30 s at 65°C. The relative expression of target genes was normalized to GAPDH and analyzed by ΔΔCT method and presented as fold change (FC = 2^-ΔΔ*CT*^).

Due to the nature of biological sample collection and processing, a small number of samples were not included in specific analyses because of insufficient serum or RNA quantity, or due to technical issues such as PCR amplification failure or sample quality concerns (e.g., hemolysis). These exclusions were minimal and occurred randomly across study groups. No samples were excluded based on statistical outlier criteria.

### Gene enrichment and gene ontology analysis

Gene enrichment analysis was implemented using ShinyGO 0.80 (http://bioinformatics.sdstate.edu/go/) to evaluate the functional enrichment and interaction of the selected gene network, including lncRNA S1PR1-DT (S1PR1 divergent transcript or lncRNA LISPR1), lncRNA APOA1-AS, S1PR1, ApoA1, SPHK1, and SPHK2, to relevant KEGG (Kyoto Encyclopedia of Genes and Genomes, https://www.genome.jp/kegg/) pathways, molecular functions, and biological processes. All query genes were first converted to ENSEMBL gene IDs: lncRNA LISPR1 (ENSG00000225938), lncRNA APOA1-AS (ENSG00000235910), S1PR1 (ENSG00000170989), ApoA1 (ENSG00000118137), SPHK1 (ENSG00000176170), and SPHK2 (ENSG00000063176). To analyze the interactions among the studied protein molecules, a protein–protein interaction network was designed using STRING database (https://string-db.org/).

### Statistical analysis

GraphPad Prism software^®^, version 8 (GraphPad Software Inc., San Diego, U.S.A.), was employed for the statistical analysis of data. First, the Shapiro–Wilk test was performed for normality testing of the assayed parameters. Mean, standard error of the mean (SEM), or median and range were used to represent experimental data, while frequency and percentage exemplified categorical data.

For normally distributed data, unpaired Student’s two-tailed t-test and ANOVA followed by Tukey’s post hoc test were employed, while Mann–Whitney or Kruskal–Wallis test followed by Dunn’s post hoc test were used for non-parametric data. For comparison of categorical data, Chi-square test or Fischer exact test were conducted. The significance level was taken into consideration at *P* <0.05, with a 95% confidence interval.

Spearman correlation was used to assess associations between variables. *P*-values for correlation analyses were adjusted for multiple comparisons using the Benjamini–Hochberg false discovery rate (FDR) method, with the FDR set at 5% (q <0.05). Adjusted q values were used to determine statistical significance for correlations involving multiple comparisons.

## Results

### Demographics, clinical characteristics, and MRI features of study population

**Figure 1:**
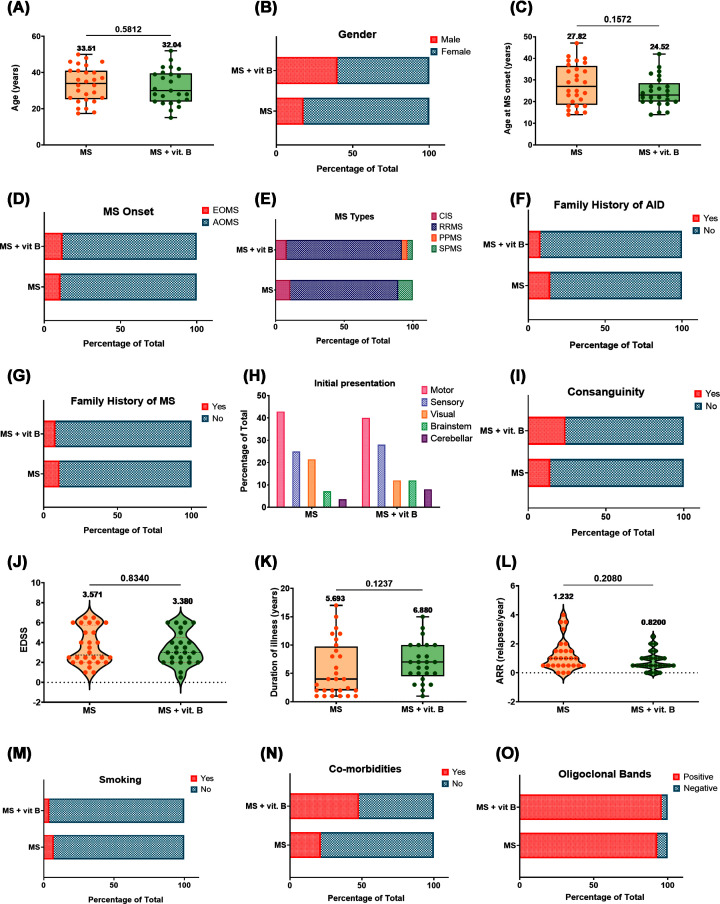
Demographic and clinical characteristics of the studied MS population (**A**) age, (**B**) gender, (**C**) age at disease onset, (**D**) type of MS according to onset, (**E**) MS types, (**F,G**) family history of AID and MS, respectively, (**H**) initial symptoms at presentation of MS, (**I**) consanguinity, (**J**) EDSS, (**K**) duration of illness, (**L**) ARR, (**M**) smoking habit of studied population, (**N**) co-morbidities, and (**O**) presence of oligoclonal bands in CSF. Differences between groups were analyzed by the Mann–Whitney test in panels (J, K, and L), whereas in panels (A and C), the two-tailed unpaired t test was used. Continuous datasets in panels (A, C, and K) were represented as box plots, whereas panels (J and L) were represented as violin plots. Box plots show all the individual data with the median as a horizontal line inside each box and the mean appears above the upper whisker. Boxes delineate 25–75th percentiles, while whiskers show the minimum and maximum values. Violin plots show the distribution and density of the individual data with the mean appearing on graph. *P*-values are indicated on the graph. Chi-square test or Fischer exact test were used in panels (B, D, E, F, G, H, I, M, N, and O). Each plot represents the data of 28 MS patients not receiving any supplements or 25 MS patients receiving vitamin B supplements. AID: autoimmune disease, AOMS: adult-onset multiple sclerosis, ARR: Annual relapse rate, n: number, CSF: cerebrospinal fluid, CIS: clinically isolated syndrome, EDSS: expanded disability status scale, EOMS: early-onset multiple sclerosis, MS: multiple sclerosis, RRMS: relapsing-remitting multiple sclerosis, SPMS: secondary progressive multiple sclerosis, PPMS: primary progressive multiple sclerosis, y: year.

The demographics and clinical characteristics of MS patients are summarized in Figure 1 and Supplementary Table S2. Mean age and sex distribution were found to be similar in the studied MS groups. Additionally, no significant differences were noted between groups in terms of age and symptoms at onset, annual relapse rate (ARR), and expanded disability status scale (EDSS) score. No clinically significant differences in symptom-related clinical characteristics were observed between the two groups at baseline. On the other hand, only 21.4% of the no-supplement group reported comorbidities compared with 48% of the vitamin B supplement group. Furthermore, no statistically significant differences have been observed in the distribution of DMTs between the study groups.

Regarding the brain and spinal cord MRIs of the study population, 89.3% of no-supplement group and 84% of vitamin B-supplement group exhibited typical MRI presentation for MS. Further, no statistically significant differences were observed in the presence of atrophy and the position of MRI lesions between the different groups. However, 25% of the no-supplement group had black holes in their MRIs compared with only 4% of the group receiving vitamin B supplementation. Moreover, in no-supplements group, 50% of MS patients showed less than 3 lesions compared with 0% in vitamin B supplement group. Whereas 25% of MS patients showed 3 to 9 MRI lesions compared with 72% of patients receiving vitamin B supplementation. As for the spinal cord MRI, 44% of vitamin B supplement group had no spinal cord lesions compared with only 7.1% of no-supplement group. The brain and spinal cord MRIs of the study population are presented in Table 1.

**Table 1:** MRI features of the study population

	No supplement	MS patients with Vitamin B Supplement	*P-value*
	*n* = 28	*n* = 25	
**MRI**			
**Typicality; n (%)**			
Typical	25 (89.3%)	21 (84%)	0.5705
Atypical	3 (10.7%)	4 (16%)	
**Black holes; n (%)**	7 (25%)	1 (4%)	0.033*
**Atrophy; n (%)**	3 (10.7%)	2 (8%)	0.7358
**Number of lesions; n (%)**			
less than 3	14 (50%)	0 (0%)	<0.0001*
3–9	7 (25%)	18 (72%)	0.0006*
10 or more	7 (25%)	7 (28%)	0.8047
**Position of lesions; n (%)**			
Juxtacortical	10 (35.7%)	13 (52%)	0.2324
Periventricular	19 (67.9%)	19 (76%)	0.5112
Brain stem	5 (17.9%)	9 (36%)	0.1348
Cerebellar	7 (25%)	9 (36%)	0.1348
Pericallosal	6 (21.4%)	4 (16%)	0.6141
**MRI spinal cord; n (%)**			
<3 segments	15 (53.6%)	7 (28%)	0.0593
≥3 segments	11 (39.3%)	7 (28%)	0.3865
none	2 (7.1%)	11 (44%)	0.0019*

Data are represented as numbers (%). *P-values* less than 0.05 were considered statistically significant and annotated with an asterix (*). MS: multiple sclerosis, MRI: magnetic resonance imaging, and *n*: number.

### Association of vitamin B supplementation with lipid profile in MS patients

**Figure 2:**
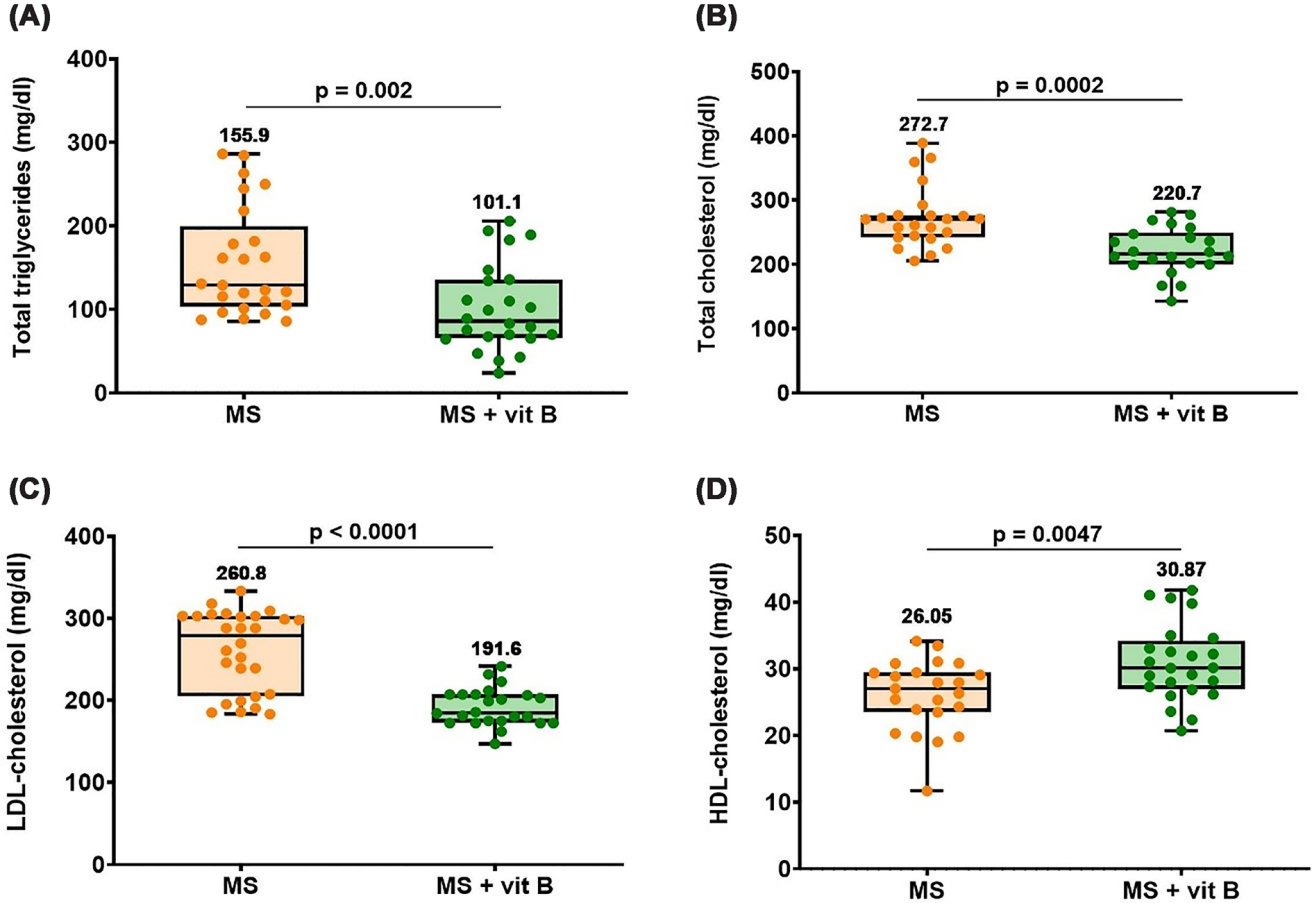
Lipid profile in MS patients according to vitamin B supplementation status (**A**) Total triglycerides, (**B**) total cholesterol, (**C**) LDL-cholesterol, and (**D**) HDL-cholesterol concentrations. Differences between groups were analyzed by the Mann–Whitney test in panels (A, B, and C), whereas in panel (D) the two-tailed unpaired t test was used. Box plots show all the individual data with the median as a horizontal line inside each box and the mean appearing above the upper whisker. Boxes delineate 25–75th percentiles, while whiskers show the minimum and maximum values. Each plot represents the data of 24–28 MS patients not receiving any supplements or 22–25 MS patients receiving vitamin B supplements. *P*-values are indicated on the graph. HDL: high-density lipoprotein and LDL: low-density lipoprotein, MS: multiple sclerosis.

As shown in Figure 2, MS patients receiving vitamin B supplementation exhibited a more favorable lipid profile compared with non-supplemented patients, with significantly decreased total triglycerides, total cholesterol, and LDL cholesterol (Figure 2A–C). Whereas vitamin B supplementation group was associated with a significantly elevated HDL cholesterol when compared with MS patients with no supplementation (Figure 2D).

### Association of vitamin B supplementation with ApoA1/APOA1-AS axis in MS patients

**Figure 3:**
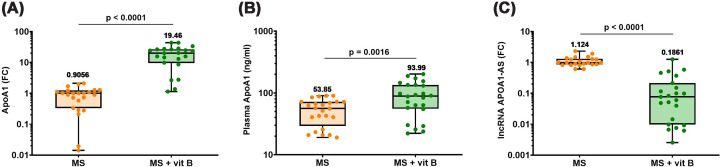
ApoA1/APOA1-AS axis in MS patients according to vitamin B supplementation status (**A**) ApoA1 gene expression, (**B**) plasma ApoA1 concentration, (**C**) APOA1-AS expression. Differences between groups were analyzed by unpaired t-test in panels (A and B), whereas in panel (C), the Mann–Whitney test was used. Box plots show all the individual data with the median as a horizontal line inside each box and the mean appearing above the upper whisker. Boxes delineate 25–75th percentiles, while whiskers show the minimum and maximum values. Each plot represents the data of 24–28 MS patients not receiving any supplements or 22–25 MS patients receiving vitamin B supplements.* P*-values are indicated on the graph. ApoA1: apoprotein A1, APOA1-AS: long non-coding RNA apoprotein A1- antisense, FC: fold change in expression, MS: multiple sclerosis.

MS patients receiving vitamin B supplementation showed significantly higher ApoA1 gene expression and plasma levels compared with non-supplemented patients (Figure 3A,B). In addition, vitamin B-supplemented MS group was accompanied with significantly lower APOA1-AS expression compared with no-supplement group, as shown in Figure 3C.

### Association of vitamin B supplementation with SPHK1/SPHK2 and S1P in MS patients

**Figure 4:**
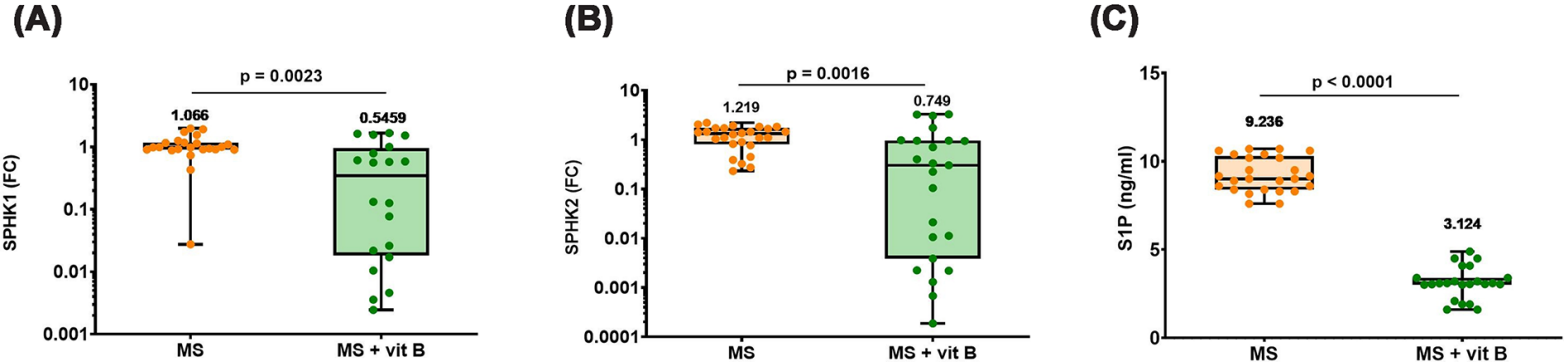
SPHK1/SPHK2 and S1P in MS patients according to vitamin B supplementation status (**A**) SPHK1, (**B**) SPHK2 gene expressions, and (**C**) plasma levels of S1P. Differences between groups were analyzed by the Mann–Whitney test in panels (A and B), while in panel (C), the unpaired t test was used. Box plots show all the individual data with the median as a horizontal line inside each box and the mean appearing above the upper whisker. Boxes delineate 25–75th percentiles, while whiskers show the minimum and maximum values. Each plot represents the data of 24–28 MS patients not receiving any supplements or 22–25 MS patients receiving vitamin B supplements. *P-*values are indicated on the graph. S1P: sphingosine 1-phosphate, SPHK1: sphingosine kinase-1, SPHK2: sphingosine kinase-2, FC: fold change in expression, and MS: multiple sclerosis.

MS patients receiving vitamin B supplementation showed significantly lower expression levels of SPHK1 and SPHK2 compared with non-supplemented patients, as shown in Figure 4A,B. Similarly, the levels of S1P in plasma of MS patients were higher than those receiving vitamin B supplements, as illustrated in Figure 4C.

### Association of vitamin B supplementation with S1PR1/LISPR1 axis of MS patients

**Figure 5:**
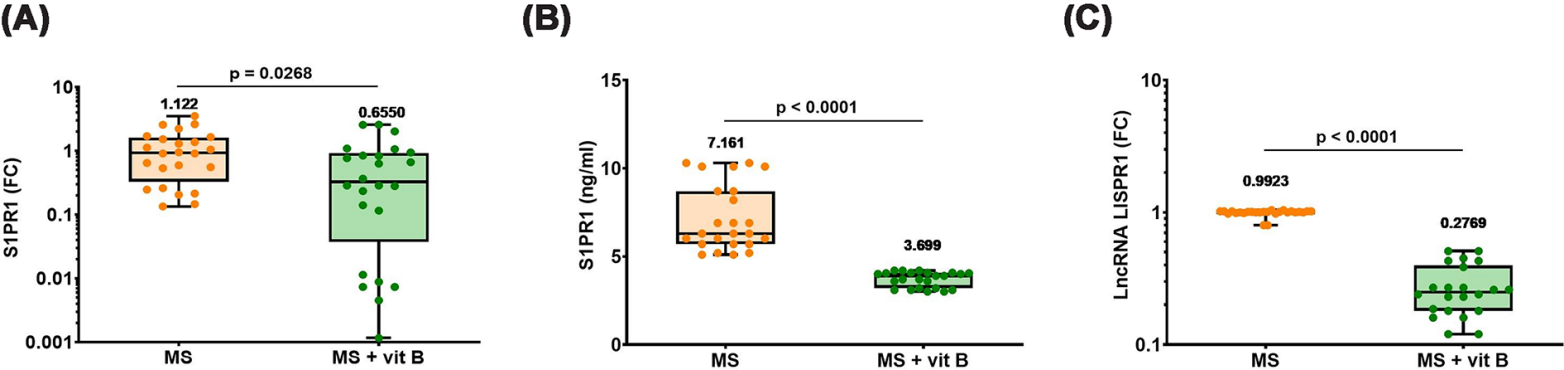
S1PR1/LISPR1 axis in MS patients according to vitamin B supplementation status (**A**) S1PR1 gene expression, (**B**) plasma levels of S1PR1, and (**C**) lncRNA LISPR1 expression. Differences between groups were analyzed by the Mann–Whitney test. Box plots show all the individual data with the median as a horizontal line inside each box and the mean appearing above the upper whisker. Boxes delineate 25–75th percentiles, while whiskers show the minimum and maximum values. Each plot represents the data of 24–28 MS patients not receiving any supplements or 22–25 MS patients receiving vitamin B supplements. *P*-values are indicated on the graph. lncRNA LISPR1: long intergenic noncoding RNA antisense to sphingosine-1-phosphate receptor-1, S1PR1: sphingosine-1-phosphate receptor-1, FC: fold change in expression, MS: multiple sclerosis.

MS patients receiving vitamin B supplementation exhibited significantly lower expression and plasma levels of S1PR1, as well as lower LISPR1 expression, compared with non-supplemented patients, as illustrated in Figure 5A–C, respectively.

### Correlations between lncRNAs APOA1-AS and LISPR1 and other parameters among MS patients

**Figure 6:**
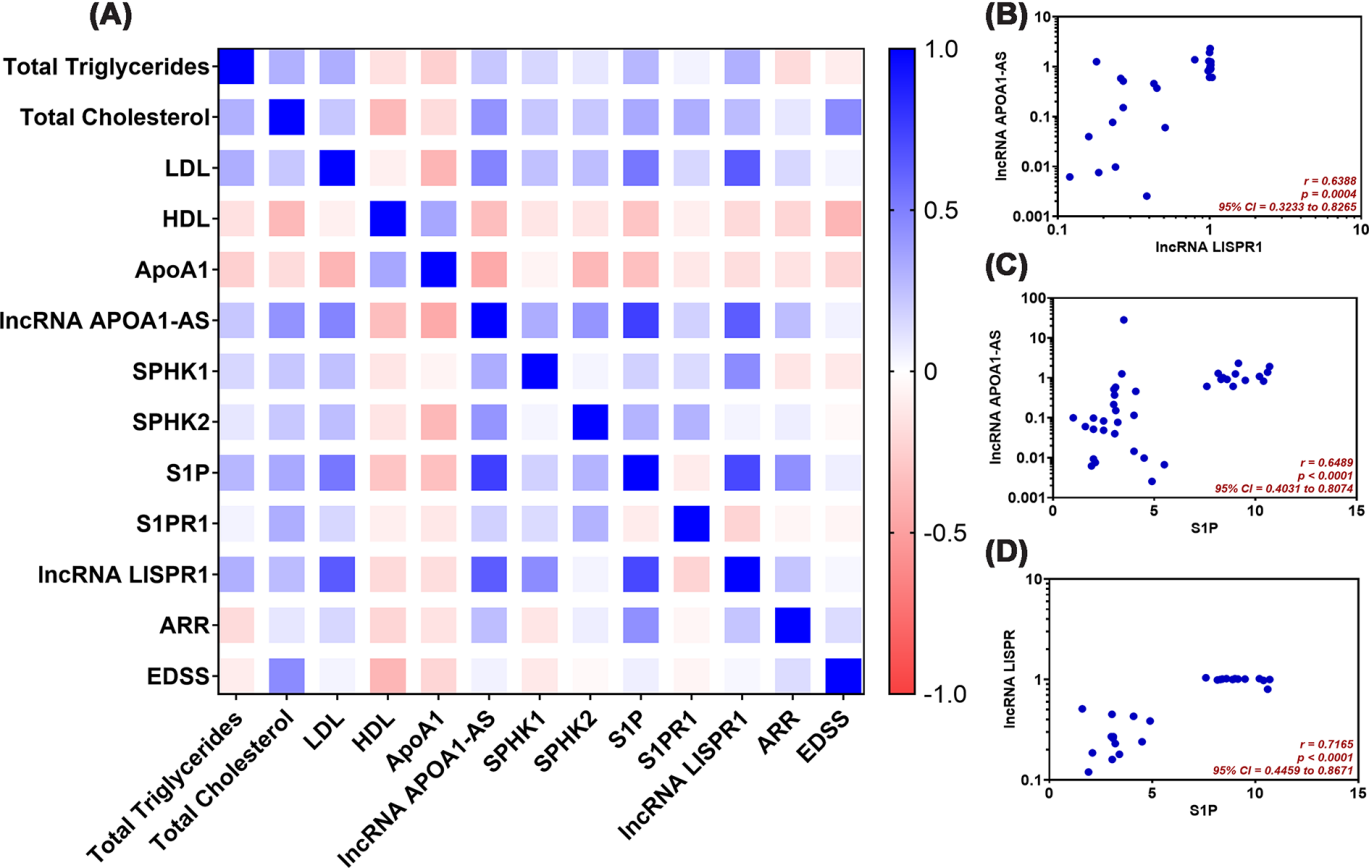
Heatmap of correlations and descriptive correlational analyses (**A**) Heatmap summarizing the correlations between lncRNAs APOA1-AS and LISPR1 and other parameters among MS patients. Correlations between the parameters were calculated using Spearman’s rank-order correlation analysis. The scale on the right of the heatmap represents the correlation coefficients (r). The color gradient spans from r = −1.0 to r = 1.0, where 1.0 indicates a perfect positive correlation (blue) and −1.0 signifies a perfect negative correlation (red), while white indicates no correlation (r = 0). (**B**) correlation between lncRNA APOA1-AS and lncRNA LISPR1, (**C**) correlation between lncRNA APOA1-AS and S1P, and (**D**) correlation between lncRNA LISPR1 and S1P. ApoA1: apolipoprotein A1, ARR: annual relapse rate, EDSS: expanded disability status scale, HDL: high-density lipoprotein, lncRNA APOA1-AS: long noncoding RNA apolipoprotein A-1 antisense RNA, lncRNA LISPR1: long intergenic noncoding RNA antisense to sphingosine-1-phosphate receptor-1, S1P: sphingosine 1-phosphate, S1PR1: sphingosine-1-phosphate receptor-1, SPHK1: sphingosine kinase-1, SPHK2: sphingosine kinase-2.

Several significant positive correlations were identified, with the most significant being between lncRNA APOA1-AS and S1P (Spearman r = 0.7502, *P* = 0.00001, q = 6.619 × 10^−5^), followed by lncRNA LISPR1 and S1P (Spearman r = 0.7165, P = 0.00004, q = 2.496 × 10^−4^), and lncRNA LISPR1 and lncRNA APOA1-AS (Spearman r = 0.6388, P = 0.00044, q = 0.001), as illustrated in Figure 6 and Supplementary Table S3.

Additionally, statistically significant negative correlations were also found between HDL and EDSS (Spearman r = −0.3858, P = 0.00739, q = 0.048), and between lncRNA APOA1-AS with ApoA1 (Spearman r = −0.44807, P = 0.00202, q = 0.013) and HDL (Spearman r = −0.34179, P = 0.02156, q = 0.013), as illustrated in Figure 6A and Supplementary Table S3.

### Gene ontology analysis

**Figure 7:**
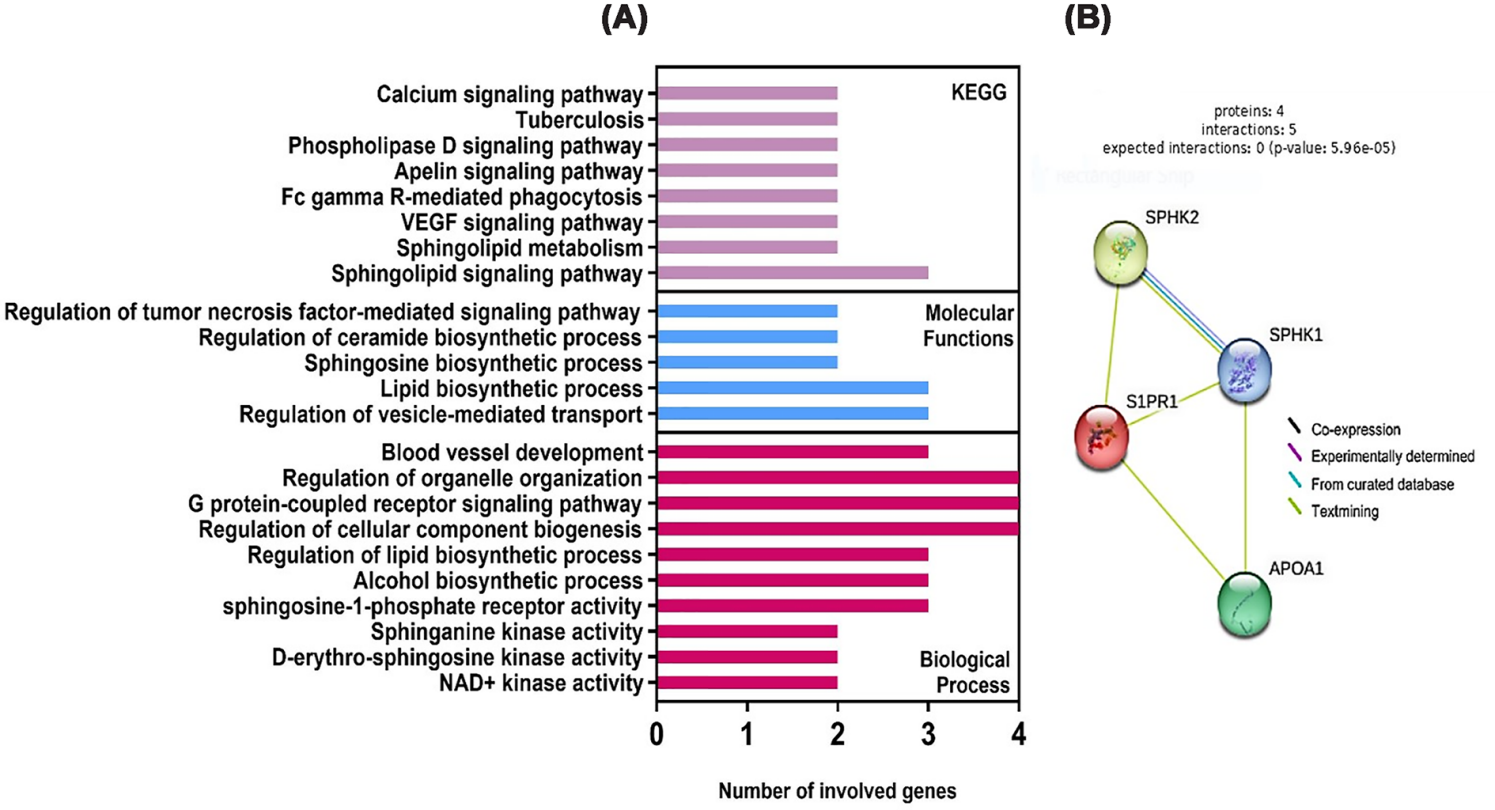
Functional enrichment and protein- protein interaction network analysis of the studied differentially expressed genes. (**A**) STRING-enriched sets of the top 10 intersected pathways involving our studied genes in KEGG pathways, molecular function domain, and biological processes domains. (**B**) Protein–protein interactions of differentially expressed genes. ApoA1: apoprotein A1, KEGG: Kyoto encyclopedia of genes and genomes, S1PR1: sphingosine-1-phosphate receptor-1, SPHK1: sphingosine kinase-1, SPHK2: sphingosine kinase-2.

Gene enrichment analysis revealed that the studied gene set is significantly involved in pathways related to sphingolipid signaling, lipid metabolism, and immune regulation (Figure 7A). The most enriched KEGG pathways included sphingolipid signaling and sphingolipid metabolism, along with VEGF signaling and Fc gamma receptor-mediated phagocytosis. Analysis of molecular functions demonstrated enrichment in sphingosine-1-phosphate receptor activity and sphingosine kinase activity.

Biological process enrichment further highlighted regulation of lipid biosynthetic processes, sphingosine and ceramide biosynthesis, and G protein-coupled receptor signaling pathways, in addition to processes related to tumor necrosis factor signaling and blood vessel development. PPI network analysis demonstrated strong connectivity among the studied proteins, with SPHK1, SPHK2, and S1PR1 forming central interaction hubs (Figure 7B).

## Discussion

The current study describes associations between long-term vitamin B1/B6/B12 supplementation and different pathways implicated in MS pathogenesis. It pointed out for the first time significantly lower expression levels of SPHK1/SPHK2 as well as lncRNA LISPR1/S1PR1/S1P in serum of MS patients receiving vitamin B1/B6/B12 supplements compared with those without supplementation. Additionally, it explored the possible role of vitamin B1/B6/B12 supplements in modulating lipid metabolism and ApoA1/lncRNA APOA1-AS gene expression levels in MS patients.

MS is characterized by CNS inflammation and demyelination leading to neurological disability [40,41]. While previous studies have demonstrated the critical role of B vitamins in normal neuronal function and remyelination [12,42–44], our results extend this knowledge by suggesting that B-vitamin supplementation may affect molecular pathways linked to both inflammation and lipid metabolism. Given that lower B-vitamin levels, particularly vitamins B1, B6, and B12, have been reported in MS patients and associated with neurological manifestations overlapping with MS [12,44,45], our results support a modulatory role of B-vitamins supplementation in disease-related pathways.

Beyond neurological effects, our results suggest a link between vitamin B1/B6/B12 supplementation and improved lipid profile. Supplemented patients exhibited lower serum triglycerides, total and LDL cholesterol, besides higher HDL-cholesterol levels. Our results are in harmony with previous reports [46–49] and may be particularly relevant given the increased cardiovascular risk observed in MS patients [50,51].

The role of vitamin B12 in lipid metabolism has been previously explained by several mechanisms, since vitamin B12 is required for S-adenosylmethionine (SAM) synthesis that is important for lipid metabolism [48]. Also, vitamin B12 supplementation has been associated with increased HDL cholesterol levels by potentially improving hepatic metabolism and autoxidation [52].

On the other hand, deficiency of vitamin B12 has been revealed to be associated with accumulation of methylmalonyl-CoA that may inhibit carnitine palmitoyl transferase-1 (CPT-1) and consequently represses fatty acid oxidation and enhances lipogenesis [53]. Furthermore, vitamin B9/B12 deficiency has been linked to high total homocysteine concentrations, thus potentially affecting phospholipid metabolism; which, in turn, may contribute to an aberrant lipid profile by increasing the hepatic output of very low-density lipoprotein (VLDL) [46,54]. Also, deficiency of vitamin B12 has been demonstrated to be associated with increased transcription of several genes required for cholesterol synthesis, including low-density lipoprotein receptor (LDLR), sterol regulatory element-binding protein-2 (SREBF2), 3-hydroxy-3-methylglutaryl-CoA reductase (HMGCR), and sterol regulatory element binding protein-1 (SREBF1) [53].

At the molecular level, our findings indicate that B-vitamin supplementation might be associated with higher ApoA1 gene expression and protein concentration along with down-regulation of lncRNA APOA1-AS, compared with MS patients without supplementation. Given that ApoA1, the most abundant protein component of HDL, has been linked to neuroprotection (Meyers et al., 2014; Samangooei et al., 2022) and better treatment response to interferon-β (IFNβ) therapy in MS patients (Gandhi et al., 2010), these results may suggest a beneficial regulatory effect. Such results agree with an earlier report of a prospective nested case-referent study that demonstrated a positive correlation between vitamin B12/B9 levels and ApoA1 level (Söderström et al., 2013). However, conflicting findings in the literature [55,56] indicate that such effects may be influenced by the nature and dose of vitamin B supplements in addition to the length of the intervention period.

Referring to sphingosine kinases; SPHK1 and SPHK2 are key enzymes responsible for S1P production [57,58]. S1P, an active phospholipid, regulates a variety of cellular responses related to immunology, heart rate, smooth muscle tone, and endothelial barrier function through its interaction with S1P receptors [59]. S1P contributes significantly to MS development and experimental autoimmune encephalomyelitis (EAE) in mice by binding to S1PR1, which is expressed on the surface of lymphocytes and is crucial in controlling lymph node egress and promoting inflammatory cell migration into the CNS [60].

There are contrasting reports about SPHK levels in MS. Ghaiad et al. (2020) reported that SPHK1 and SPHK2 levels were up-regulated in Egyptian RRMS patients [61]. But Luo et al. (2017) showed significantly higher levels of only SPHK1 in a murine model of MS with insignificant change in SPHK2 levels [62]. Additionally, SPHK1 mRNA and protein expression were up-regulated in EAE rats group compared with control group [63]. As for SPHK2, it has been reported that SPHK2 is necessary for remyelination following cuprizone intoxication in mice [64].

For the first time, our study demonstrated that vitamin B1/B6/B12 supplementation to MS patients was associated with lower gene expression of both SPHK1 and SPHK2, decreasing S1P level in comparison with MS patients not receiving any supplements. These findings align with a prior report showing that vitamin B6 prevented higher inflammation in EAE model [65] by increasing the activity of S1P lyase resulting in reduction of S1P accumulation. Moreover, it has been observed that vitamin B6 level is inversely correlated with S1P level in sphingosine lyase insufficiency syndrome patients [66] and in IL10^−/−^ mouse model of irritable bowel syndrome (IBD) [67] by increasing S1P lyase activity. Recently, Jonnalagadda et al. (2023) reported that vitamin B12 restriction eliminated the efficacy of FTY720 (fingolimod), a sphingosine analog and S1P receptor modulator authorized for MS treatment efficacy [68].

Targeting S1P/S1PRs axis has been suggested as a possible treatment for a lot of immune-mediated conditions, including MS, Crohn’s disease, psoriasis, atopic dermatitis, ulcerative colitis, rheumatoid arthritis, and systemic lupus erythematosus (SLE) [69,70]. Several research studies conducted in mouse models of MS had revealed that S1PR1 blockade inhibits cytokine amplification and immune cell recruitment that are considered the primary therapeutic targets in MS [59,71,72].

Interestingly, lncRNA LISPR is positioned closely to S1PR1 gene and shares its promoter region as well. Josipovic et al. (2018) showed that LISPR1 and S1PR1 expressions were decreased in human pulmonary diseases such as COPD. Additionally, LISPR1 knockdown was demonstrated to attenuate S1PR1 expression, which in turn decreased S1PR1-dependent signaling of S1P [35].

To our knowledge, this research is the first to investigate the potential involvement of lncRNA LISPR1 in MS pathogenesis in relation to S1PR1 signaling pathway. Our results showed that vitamin B1/B6/B12 supplementation in MS patients was associated with lower LISPR1 and S1PR1 expression with a simultaneous decline in S1P protein level compared with those without supplements. Additionally, our study suggested for the first time that lncRNA APOA1-AS expression might be directly correlated with S1P and lncRNA LISPR1 among the vitamin B1/B6/B12-supplemented group, while being inversely correlated with ApoA1 and HDL cholesterol. On the other hand, in our study population, lncRNA LISPR1 expression appeared to be directly interconnected with S1P levels. Altogether, our findings offer a novel perspective on the potential associations between vitamin B1/B6/B12 supplementation, lipid profile regulation, and sphingosine-related pathways in MS patients.

The gene enrichment findings further support the biological relevance of the selected targets, as the identified pathways were predominantly related to sphingolipid signaling, lipid metabolism, and inflammatory processes. In particular, the enrichment of sphingosine biosynthetic processes is consistent with the central role of the S1P/S1PR1 axis in immune cell trafficking and neuroinflammation in MS. Additionally, the involvement of vascular-related pathways such as VEGF signaling and blood vessel development may reflect the contribution of BBB dysfunction to disease progression.

Although our measured parameters may be affected by variable factors such as dietary habits, physical activity, body mass index, metabolic status, systemic inflammation, disease severity, and the use of DMTs, our supplemented and non-supplemented groups were recruited from the same clinical center and geographical area and exhibited comparable demographic and clinical characteristics. Therefore, environmental, lifestyle, and treatment-related factors are unlikely to fully explain these observed differences, supporting a potential, but not strictly causal, contribution of vitamin B1/B6/B12 supplementation to the molecular and metabolic changes observed in supplemented MS patients.

Furthermore, in the present study, patients receiving sphingosine-1-phosphate receptor modulators were excluded to avoid direct pharmacological interference with S1P signaling pathways. While other DMTs were not controlled for or stratified during recruitment, no statistically significant differences were observed in their distribution between the study groups, which reduces, but does not eliminate, their potential confounding effect.

Different DMTs are known to modulate immune responses, lipid metabolism, and gene expression, and thus may have contributed, at least in part, to the observed molecular and biochemical differences. Although DMT regimens were heterogeneous, their distribution did not significantly differ between groups, which may reduce—but not eliminate—their confounding effect. Importantly, the consistent directionality of changes observed across multiple related pathways, including lipid profile, ApoA1/APOA1-AS axis, and S1P signaling components, suggests a coordinated biological pattern that may not be solely attributable to differences in DMT use.

Although a higher frequency of comorbidities was observed in the vitamin-supplemented group, these conditions were heterogeneous and primarily included non-metabolic states (e.g., osteoporosis, epilepsy, depression, rheumatic heart disease, seasonal asthma, and peptic ulcer). Such conditions are unlikely to exert sustained effects on lipid metabolism or apolipoprotein regulation and therefore are not expected to significantly confound the observed biochemical and molecular outcomes.

### Limitations and recommendations

While the present study may provide novel preliminary insights into the molecular pattern associated with vitamin B1/B6/B12 supplementation in MS patients, several limitations should be acknowledged. First, the observational nature of the study limits causal inference, and supplementation was not randomly assigned, but rather based on clinical decision-making by treating physicians. As such, bias may have been introduced through physician prescribing patterns or unmeasured differences between supplemented and non-supplemented groups. Furthermore, supplement adherence was self-reported and not objectively verified, which may affect the interpretation of the findings.

Additionally, the sample size, while adequate for preliminary exploratory analyses, may not capture the full heterogeneity of the MS population, including disease subtypes and duration. Importantly, the present study was designed as a targeted, hypothesis-driven clinical investigation rather than a comprehensive mechanistic analysis, and therefore does not provide definitive functional or causal validation of the observed molecular associations.

A further limitation of the present study is the lack of objective verification of vitamin B supplementation adherence. Although patients in the supplemented group reported regular intake of the prescribed vitamins, compliance was not confirmed through measurement of circulating vitamin levels or formal adherence monitoring. Additionally, while patients in the non-supplemented group were not prescribed vitamin B supplements, the possibility of unreported over-the-counter (OTC) supplement use cannot be entirely excluded. Therefore, the observed associations should be interpreted with caution, and future studies incorporating objective assessment of vitamin status are warranted to strengthen causal inference.

Future research should utilize randomized controlled trial designs to validate these findings with better control for bias and adherence. Including larger, diverse cohorts with longitudinal follow-up and integrating functional and omics-based analyses will help clarify the clinical and mechanistic relevance of B-vitamin supplementation in MS and further dissect the mechanistic roles of the studied lncRNAs and sphingolipid mediators.

## Conclusion

**Figure 8:**
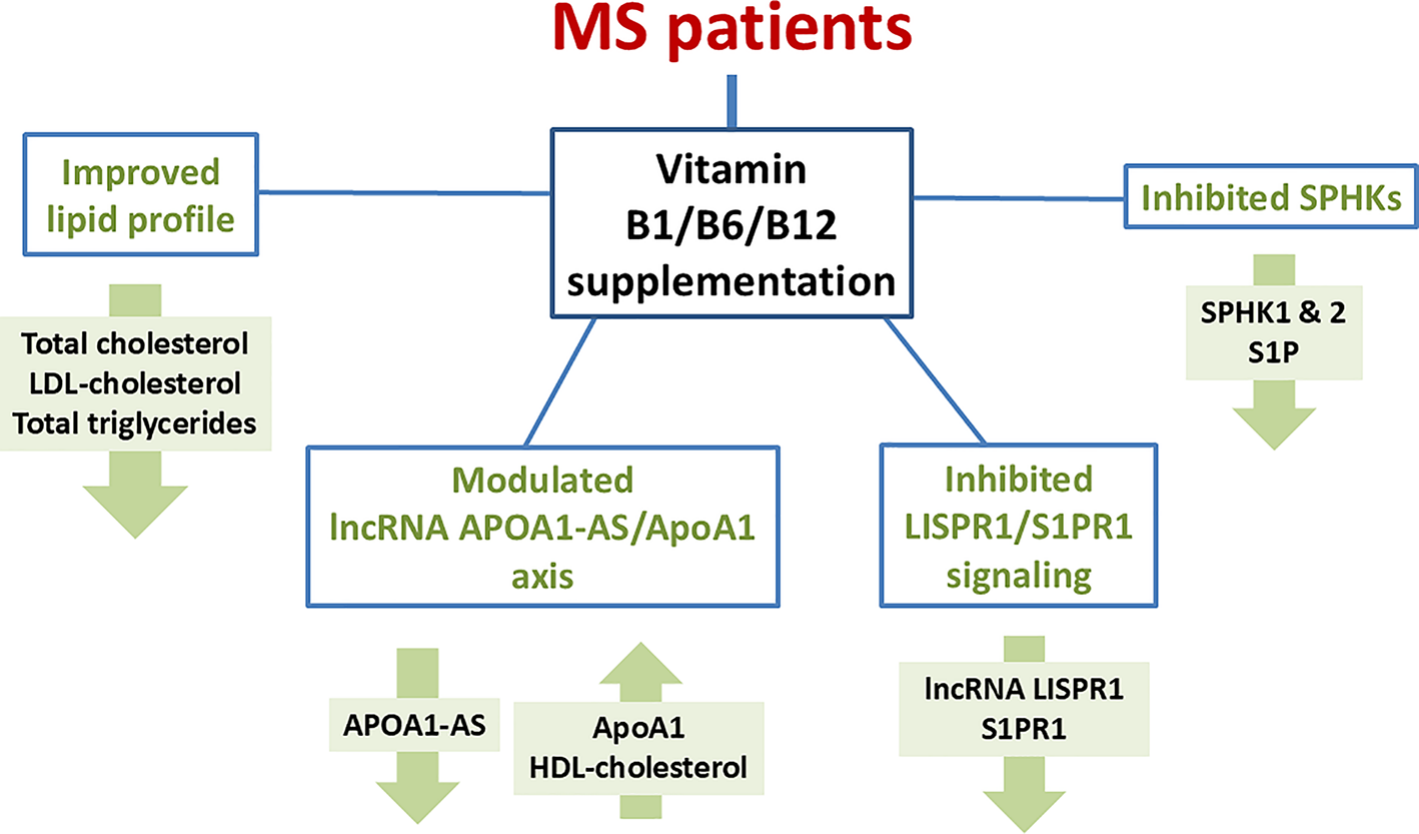
Schematic presentation of the study conclusion

In conclusion, we have observed that long-term vitamin B1/B6/B12 supplementation might not only be associated with lower SPHK1/SPHK2 and lncRNA LISPR1/S1PR1 expression levels, in addition to being most likely associated with reduced S1P concentration, and potentially attenuating S1PR1-dependent signaling of S1P, but it also seems to be associated with alterations in lipid metabolism and normalization of ApoA1/lncRNA APOA1-AS expression in MS patients, as summarized in Figure 8.

## Supplementary Material

Supplementary Tables S1-S3

## Data Availability

The datasets used and/or analyzed during the current study are available from the corresponding author on reasonable request.

## Abbreviations

**ApoA1**: Apoprotein A1
**ARR**: Annual relapse rate
BBB: Blood-brain barrier
**CPT-1**: Carnitine palmitoyl transferase-1
CNS: Central nervous system
COPD: Chronic obstructive pulmonary disease
**DMTs**: Disease-modifying therapies
**EAE**: Experimental autoimmune encephalomyelitis
**EDSS**: Expanded disability status scale
**FC**: Fold change in expression
FDR: False discovery rate
GAPDH: Glyceraldehyde-3-phosphate dehydrogenase
**HDL**: High-density lipoprotein
HMGCR: 3-hydroxy-3-methylglutaryl-CoA reductase
**IBD**: Irritable bowel syndrome
**IFNβ**: Interferon-β
KAMSU: Kasr Al-Ainy Multiple Sclerosis Research Unit
**KEGG**: Kyoto encyclopedia of genes and genomes
**LDL**: Low-density lipoprotein
LDLR: Low-density lipoprotein receptor
**lncRNA APOA1-AS**: Long noncoding RNA apolipoprotein A-I antisense RNA
**lncRNA LISPR1**: Long intergenic noncoding RNA antisense to sphingosine-1-phosphate receptor-1
**MCP-1**: Monocyte chemotactic protein 1
**MIP-1**: Macrophage inflammatory protein 1
**MS**: Multiple sclerosis
MRI: Magnetic resonance imaging
OTC: Over-the-counter
REC-FOPCU: Research Ethics Committe for experimental and clinical studies at the Faculty of Pharmacy of Cairo University
**S1P**: Sphingosine-1-phosphate
**S1PR1**: Sphingosine-1-phosphate receptor-1
**SAM**: S-adenosylmethionine
SEM: Standard error of the mean
**SLE**: Systemic lupus erythematosus
**SPHK1**: Sphingosine kinase1
**SPHK2**: Sphingosine kinase2
SREBF1: Sterol regulatory element binding protein-1
SREBF2: Sterol regulatory element binding protein-2
**VCAM-1**: Vascular cell adhesion molecule-1
**VLDL**: Very low-density lipoprotein
