# Supplementary material for: Association of vitamin B1/B6/B12 supplementation with sphingosine-1-phosphate signaling and its receptors in multiple sclerosis patients: relevance to LISPR1 and APOA1-AS
Source: Biosci Rep. 2026 May 21;46(6):BSR20260065. doi: 10.1042/BSR20260065 (PMC13199797; doi:10.1042/BSR20260065)
Supplement: Supplementary Tables S1-S3 [file BSR-2026-0065_supp.pdf]

Effect of vitamin B1/B6/B12 supplementation on sphingosine 1-phosphate and its receptors in Multiple Sclerosis patients: Implications of lncRNAs LISPR1 and APOA1-AS

Noha A. Mehana [a#] , Heba R. Ghaiad [a*#], Mohammed M. Nooh [a] , Mai A. Amer [b], Lobna Talaat El-Ghoneimy [c], Maheera H. Safwat [a*]

Supplementary table (1): Primer sequences used in qRT-PCR.

Gene	Forward primer (5'-3')	Reverse primer (5'-3')
**GAPDH**	ACCTTGTGTCCCTCAATATGGT	GTACTCAGCGCCAGCATCG
**lncRNA APOA1-AS**	ATGCTGGTCACTTCAGTCCC	AGGGGATTGGTTATGAGGCT
**SPHK1**	ATCTAACTCGAGGTGCTCGC	AGTAGGGACGCGTTTGTCAG
**SPHK2**	GGCCTTTGTTACGCGTGTTAG	TGGGCCTGTCTCA TCCATTG
**S1PR1**	GGGAGCAATAACTTCCGCCT	AAGACCGTGGTGCAGAAGAG
**lncRNA LISPR1**	CTGCGTGACAAGCTCAGGACAG	GAGTTCACATCTGCCGGGATGG

ApoA1: apolipoprotein A1, lncRNA APOA1-AS: long noncoding RNA apolipoprotein A-1 antisense RNA, GAPDH: glyceraldehyde 3-phosphate dehydrogenase, lncRNA LISPR1: long intergenic noncoding RNA antisense to sphingosine-1-phosphate receptor-1, S1PR1: sphingosine-1-phosphate receptor-1 ,SPHK1: sphingosine kinase-1, SPHK2: sphingosine kinase-2.

Effect of vitamin B1/B6/B12 supplementation on sphingosine 1-phosphate and its receptors in Multiple Sclerosis patients: Implications of lncRNAs LISPR1 and APOA1-AS

Noha A. Mehana [a#] , Heba R. Ghaiad [a*#], Mohammed M. Nooh [a] , Mai A. Amer [b], Lobna Talaat El-Ghoneimy [c], Maheera H. Safwat [a*]

Supplementary Table (2): Demographics and clinical characteristics of the study population.

		MS patients	MS patients on Vitamin B Supplement	*P value*
		n = 28	n = 25	
**Age; median (range), y**		34 (17 - 50)	30 (15 - 52)	0.5812
**Sex; female:male, n (ratio)**		23:5 (4.6:1)	15:10 (1.5:1)	0.1257
**Age at onset; median (range), y**		27 (14 - 47)	23 (14 - 42)	0.1572
**Onset; n (%)**				
	EOMS	3 (10.7%)	3 (12%)	>0.9999
	AOMS	25 (89.3%)	22 (88%)	
**Consanguinity; n (%)**		4 (14.3%)	6 (24%)	0.4878
**Family history of autoimmune diseases; n (%)**		4 (14.3%)	2 (8%)	0.6718
**Family history of MS; n (%)**		3 (10.7%)	2 (8%)	>0.9999
**Symptoms at onset; n, (%)**				
	Sensory	7 (25%)	7 (28%)	0.8177
	Motor	12 (42.9%)	10 (40%)	
	Visual	6 (21.4%)	3 (12%)	
	Brain stem	2 (7.1%)	3 (12%)	
	Cerebellar	1 (3.6%)	2 (8%)	
**Clinical Subtypes of MS; n (%)**				
	CIS	3 (10.7%)	2 (8%)	0.56
	RRMS	22 (78.6%)	21 (84%)	
	PPMS	0 (0%)	1 (4%)	
	SPMS	3 (10.7%)	1 (4%)	
**EDSS; median (range)**		2.5 (1 - 6.5)	3 (0.5 - 6)	0.8186
**Illness Duration; median (range), y**		4 (1 - 17)	6.5 (1 - 15)	0.1305
**Relapses in last 2 years; median (range)**		2 (0 - 8)	1 (0 - 5)	0.3944
**ARR; median (range)**		1 (0 - 4)	0.5 (0 - 2.5)	0.208

Effect of vitamin B1/B6/B12 supplementation on sphingosine 1-phosphate and its receptors in Multiple Sclerosis patients: Implications of lncRNAs LISPR1 and APOA1-AS

Noha A. Mehana [a#] , Heba R. Ghaiad [a*#], Mohammed M. Nooh [a] , Mai A. Amer [b], Lobna Talaat El-Ghoneimy [c], Maheera H. Safwat [a*]

		MS patients	MS patients on Vitamin B Supplement	*P value*
		n = 28	n = 25	
**Smoking; n (%)**		2 (7.1%)	1 (4%)	>0.9999
**Comorbidities (non-metabolic); n (%)**		6 (21.4%)	12 (48%)	***0.0492 ****
**Contraception; n (%)**		2 (7.1%)	2 (8%)	0.9061
**Positive oligoclonal band in CSF; n (%)**		26 (92.9%)	24 (96%)	>0.9999
**Disease-modifying therapies (DMTs); n (%)**				
	**Corticosteroids**	25 (89.3%)	23 (92%)	0.7358
	**Immunosuppressants**	12 (42.9%)	8 (32%)	0.4156
	**β-interferon**	20 (71.4%)	22 (88%)	0.1375

Data are represented as numbers (range) or (%). P values less than 0.05 was considered statistically significant and annotated with an asterix (*). Some variables (e.g., symptoms at onset, clinical subtypes, and treatments) are not mutually exclusive; therefore, percentages may not sum to 100%. *ARR: Annual relapse rate, n: number, CSF: cerebrospinal fluid, CIS: Clinically isolated syndrome, DMTs: disease-modifying therapies, EDSS: expanded disability status scale, RRMS: relapsing-remitting multiple sclerosis, SPMS: Secondary progressive multiple sclerosis, PPMS: primary progressive multiple sclerosis and y: year.*

		**Total Tri-glycerides**	**Total Cholesterol**	**LDL**	**HDL**	**ApoA1**	**lncRNA APOA1-AS**	**SPHK1**	**SPHK2**	**S1P**	**S1PR1**	**lncRNA LISPR1**	**ARR**	**EDSS**
**Total Tri-glycerides**	***r***		**0.30580**	**0.31746**	-0.15981	-0.24727	0.21793	0.15343	0.09261	0.27932	0.04598	0.30701	-0.19017	-0.09432
	p-		**0.04107**	**0.02624**	0.28325	0.09019	0.15041	0.32596	0.53129	0.16701	0.75634	0.12712	0.19060	0.51915
**Total Cholesterol**	***r***	**0.30580**		0.21817	**-0.36952**	-0.18361	**0.42172**	0.21942	0.21181	0.33470	**0.31449**	0.26144	0.09262	**0.45301**
	p-	**0.04107**		0.14993	**0.01248**	0.22731	**0.00436**	0.15744	0.16249	0.09466	**0.03538**	0.19702	0.54508	**0.00178**
**LDL**	***r***	**0.31746**	0.21817		-0.08136	**-0.38766**	**0.48131**	0.24132	0.25224	**0.53295**	0.15634	**0.64545**	0.15357	0.04174
	p-	**0.02624**	0.14993		0.58666	**0.00648**	**0.00082**	0.11901	0.08038	**0.00506**	0.28861	**0.00037**	0.27706	0.76890
**HDL**	***r***	-0.15981	**-0.36952**	-0.08136		**0.34335**	**-0.34179**	-0.13600	-0.14078	-0.30393	-0.08639	-0.19633	-0.21982	**-0.38587**
	p-	0.28325	**0.01248**	0.58666		**0.01814**	**0.02156**	0.38454	0.35071	0.13117	0.56368	0.33641	0.13763	**0.00739**
**ApoA1**	***r***	-0.24727	-0.18361	**-0.38766**	**0.34335**		**-0.44807**	-0.05890	**-0.37340**	-0.33265	-0.12101	-0.17372	-0.15156	-0.21740
	p-	0.09019	0.22731	**0.00648**	**0.01814**		**0.00202**	0.70753	**0.00973**	0.09683	0.41263	0.39604	0.30380	0.13772
**lncRNA APOA1-AS**	***r***	0.21793	**0.42172**	**0.48131**	**-0.34179**	**-0.44807**		**0.31935**	**0.41380**	**0.75021**	0.17926	**0.63880**	0.25305	0.04988
	p-	0.15041	**0.00436**	**0.00082**	**0.02156**	**0.00202**		**0.03925**	**0.00472**	**0.00001**	0.23870	**0.00044**	0.09351	0.74489
**SPHK1**	***r***	0.15343	0.21942	0.24132	-0.13600	-0.05890	**0.31935**		0.03579	0.17812	0.14089	**0.44783**	-0.13399	-0.11948
	p-	0.32596	0.15744	0.11901	0.38454	0.70753	**0.03925**		0.81975	0.38399	0.36749	**0.02178**	0.39166	0.44538
**SPHK2**	***r***	0.09261	0.21181	0.25224	-0.14078	**-0.37340**	**0.41380**	0.03579		0.29162	**0.29756**	0.04146	0.06499	-0.03427
	p-	0.53129	0.16249	0.08038	0.35071	**0.00973**	**0.00472**	0.81975		0.14832	**0.04222**	0.84063	0.65731	0.81518
**S1P**	***r***	0.27932	0.33470	**0.53295**	-0.30393	-0.33265	**0.75021**	0.17812	0.29162		-0.10359	**0.71647**	**0.43333**	0.06208
	p-	0.16701	0.09466	**0.00506**	0.13117	0.09683	**0.00001**	0.38399	0.14832		0.61455	**0.00004**	**0.02701**	0.76319
**S1PR1**	***r***	0.04598	**0.31449**	0.15634	-0.08639	-0.12101	0.17926	0.14089	**0.29756**	-0.10359		-0.22786	-0.05203	-0.05279
	p-	0.75634	**0.03538**	0.28861	0.56368	0.41263	0.23870	0.36749	**0.04222**	0.61455		0.26292	0.72542	0.72160
**lncRNA LISPR1**	***r***	0.30701	0.26144	**0.64545**	-0.19633	-0.17372	**0.63880**	**0.44783**	0.04146	**0.71647**	-0.22786		0.22369	0.02816
	p-	0.12712	0.19702	**0.00037**	0.33641	0.39604	**0.00044**	**0.02178**	0.84063	**0.00004**	0.26292		0.27199	0.89140
**ARR**	***r***	-0.19017	0.09262	0.15357	-0.21982	-0.15156	0.25305	-0.13399	0.06499	**0.43333**	-0.05203	0.22369		0.13711
	p-	0.19060	0.54508	0.27706	0.13763	0.30380	0.09351	0.39166	0.65731	**0.02701**	0.72542	0.27199		0.32756
**EDSS**	***r***	-0.09432	**0.45301**	0.04174	**-0.38587**	-0.21740	0.04988	-0.11948	-0.03427	0.06208	-0.05279	0.02816	0.13711	
	p-	0.51915	**0.00178**	0.76890	**0.00739**	0.13772	0.74489	0.44538	0.81518	0.76319	0.72160	0.89140	0.32756	

Supplementary Table (3): Correlations between the studied parameters in MS patients.

ApoA1: apolipoprotein A1, ARR: annual relapse rate, EDSS: expanded disability status scale, HDL: high-density lipoprotein, lncRNA APOA1-AS: long noncoding RNA apolipoprotein A-1 antisense RNA, lncRNA LISPR1: long intergenic noncoding RNA antisense to sphingosine-1-phosphate receptor-1, S1P: sphingosine 1-phosphate, S1PR1: sphingosine-1-phosphate receptor-1 ,SPHK1: sphingosine kinase-1, SPHK2: sphingosine kinase-2.
